# 

**Published:** 1918-08

**Authors:** 


### CONTENTS FOR THE AUGUST ISSUE.

EDITORIALS

A Gigantic War Work.... 33
The War and the Universities.... 35
General Gorgas.... 35
The War Calls.... 36

ORIGINAL ARTICLES

Indications for the Radiograph in the Study of Oral Conditions. By John M. Martin, M. D., Dallas, Texas.... 39

Peripheral Nerve Lesions. By Capt. W. L. Crosthwait, M. D., Waco, Texas.... 48

A Request from the State Health Department.... 56

Letter Concerning Dallas Meeting of Southwestern Medical Association.... 57

Government Experts Favor Extermination of Rats.... 58

Army News.... 59

Deaths.... 64

Book Reviews.... 65

Helpful Hints.... 66

Old Jokes That We Have Met.... 68

